# Electromyography varies by stage in inclusion body myositis

**DOI:** 10.3389/fneur.2023.1295396

**Published:** 2024-01-05

**Authors:** Tomoo Mano, Naohiko Iguchi, Nobuyuki Eura, Naoki Iwasa, Nanami Yamada, Hirosei Horikawa, Kazuma Sugie

**Affiliations:** ^1^Department of Neurology, Nara Medical University, Kashihara, Japan; ^2^Department of Rehabilitation Medicine, Nara Prefecture General Medical Center, Nara, Japan

**Keywords:** inclusion body myositis, electromyography, number of rimmed vacuoles, motor neuron disease, myogenic change, neurogenic change

## Abstract

**Introduction:**

Inclusion body myositis (IBM) is a chronic inflammatory muscle disease that is characterized by mixed myogenic and neurogenic electromyography (EMG) findings. We investigated the association between EMG findings and the IBM stage.

**Methods:**

We included consecutive patients diagnosed with IBM based on muscle biopsy and had needle EMG performed within 1 month of biopsy. Motor unit potential waveform (MUP) in EMG and pathological findings were compared between patients in early and late phases.

**Results:**

In total, 30 patients with biopsy-confirmed IBM and 254 muscles were included. The rate of abnormal discharge did not differ according to disease stage. There was a difference in the frequency of occurrence between myogenic suggestive MUP and neurogenic of biceps and flexor digitorum profundus in the late phase. Abnormal MUP was observed even in muscles without muscle weakness, and myogenic changes were predominant in biceps and gastrocnemius with muscle weakness. The biopsy findings on the contralateral side of the muscle where electromyography was performed revealed a tendency for muscles that exhibited myogenic origin to have more inflammatory cells and RV; however, the difference was not significant.

**Conclusion:**

The target muscles for EMG must be selected considering the disease stage as well. In the early stages of IBM, EMG results should be interpreted cautiously, as neurogenic suggestive pattern of MUP might also be exhibited. Contralateral electromyography findings may be helpful in selecting muscles for muscle biopsies, such as biceps and quadriceps.

## 1 Introduction

Inclusion body myositis (IBM) is an enigmatic, progressive disease of the skeletal muscles with both proximal and distal limb weaknesses, and it is characterized by muscle weakness beyond the typical pattern. Less common presentations include isolated, asymptomatic, and hyper-creatine kinasemia. The affected muscles, which become atrophic with the slow progression of the disease, are often weaker muscles such as the flexor digitorum profundus (FDP) and quadriceps.

IBM may be difficult to diagnose based on clinical findings alone. Moreover, IBM is sometimes misdiagnosed, leading to inappropriate treatment for motor neuron diseases or orthopedic disorders. Clinicians recommend electromyography (EMG) to distinguish myogenic from neurogenic disease as well as to decide the requirement for muscle biopsy. In clinical practice, EMG is central to the diagnosis and selection of patients for muscle biopsy (1). However, the misinterpretation of needle EMG can make the diagnosis more difficult. Needle EMG in myopathy typically demonstrates early recruitment of short-duration, low-amplitude, complex motor-unit potentials (MUPs) and fibrillation potentials. Up to one-third of patients with IBM may demonstrate myogenic or myogenic-like discharges (2, 3). However, IBM MUPs not only cause myogenic suggestive changes but also often exhibit neurogenic suggestive changes. Furthermore, the tendencies of each muscle finding should be understood. This requires an understanding of the duration of the disease and trends in individual muscle findings. Therefore, we investigated the EMG characteristics and disease stages of each muscle in patients with IBM.

## 2 Methods

### 2.1 Participants

The retrospective study included consecutive patients who were diagnosed with IBM based on muscle biopsy and had needle EMG performed within 1 month of the biopsy. Muscle biopsies and EMG were performed at the Department of Neurology of Nara Medical University from 2010 to 2020. Muscle strength was assessed before biopsy using grip strength and the Medical Research Council (MRC). Biopsies were not performed in the muscle where EMG was performed, and in the case of the same muscle, the contralateral muscle was targeted. We excluded patients with concomitant neuromuscular disorders that caused motor dysfunction. In all the patients, the results of the biopsies showed typical features such as inflammation and rimmed vacuoles. We divided patients into two groups using the median number of years elapsed, and categorized those below the median value as the early phase group and those above the median value as the late phase group.

### 2.2 EMG and muscle strength

All the EMG examinations were performed and reviewed by trained physicians and electromyographers with >10 years of experience. Concentric needle EMG was performed using a disposable concentric needle, with an uptake area of 0.07 mm^2^ and an EMG machine (Neuropack; Nihon Kohden, Tokyo, Japan). The bandpass filter was set from 2 to 20 kHz. The reports were reviewed for the description of spontaneous activity and morphology of MUPs in each examination. Each muscle was tested for at least five abnormalities to confirm reproducibility. Abnormal MUPs were categorized as myogenic suggestive patterns [short duration (<6 ms) and low amplitude] or neurogenic suggestive patterns [long duration (>16 ms) and/or high amplitude]. At least two blinded physicians with >10 years of experience interpreted the test results and judged whether the muscle was myogenic or neurogenic. Objective quantification of MUAP parameters has been previously explored, but its widespread implementation in clinical practice remains limited (4–7). Previous studies have reported no statistically significant differences between automated pattern analysis and purely qualitative visual evaluation of MUPs (8). Electromyography was conducted both during maximal effort and at rest, and the evaluation included the presence or absence of interference waves and denervation. However, for the purpose of this study, our focus was solely on the evaluation of the duration of MUP.

Muscle strength was assessed using the Manual Muscle Test (MMT) (9) by applying pressure to the muscle groups tested against gravity or through a range of motion for muscle groups with less than anti-gravity strength, and this assessment was administered by a trained neurologist. An MMT score of 5 indicated that the patient could complete the full range of available motion against gravity. In cases of EMG and muscle strength, this study we adopted the date of the more affected side.

### 2.3 Semi-quantitative assessment of pathology

We excluded one case with poor preservation and another with a crush injury from this analysis. To semi-quantify muscle pathology, measurements were taken in four randomly selected microscopic fields (×200). The counts included the number of inflammatory cells and with rimmed vacuoles.

The semi-quantification of inflammatory cells ranged from 0 to 5 in 100 units per microscopic field. The presence or absence of rimmed vacuoles was observed for each field of view and semi-quantified as 0–4.

### 2.4 Data analysis

All data are presented as means ± standard deviations. Differences in categorical variables were assessed using the *t*-test and Fisher’s exact test. *p* < 0.05 were considered statistically significant. Statistical analyses were performed using SPSS version 21.0 J (SPSS Japan, Tokyo, Japan).

## 3 Results

### 3.1 Patient background

We analyzed 30 consecutive patients (18 males and 12 females; mean age, 70.3 ± 5.7 years; mean disease duration, 3.4 ± 2.6 years) retrospectively, who were diagnosed with IBM using muscle biopsy. Table 1 summarizes the patient characteristics. There were 15 patients in the early group and 15 in the late group. In all the patients, the EMG examination was conducted 7.2 ± 5.1 days before the biopsy. The number of biopsy sites was consistent for both biceps and quadriceps. The selection of the biopsy site was based on a combination of image findings and physical assessments. The pathological findings revealed no significant difference in the number of inflammatory cells between the early and late stages. However, the number of rimmed vacuoles was observed to be higher in the late stages as compared to that in the early stages.

**Table 1:** Clinical features of patients with inclusion body myositis.

	Early phase (*n* = 15)	Late phase (*n* = 15)	*p*-value
Disease duration (years)	1.4 ± 1.0	5.3 ± 2.1	*p* < 0.001
Age (years)	69.4 ± 7.4	71.3 ± 8.2	N.S.
Sex (female:male)	9:11	3:7	N.S.
Serum creatine kinase	429.3 ± 238.1	476.1 ± 317.8	N.S.
Average grip strength (kg)	12.1 ± 7.4	12.3 ± 10.1	N.S.
MRC sum score	4.6 ± 0.52	4.3 ± 0.63	N.S.
Abnormal MUP occurrence rate (%)	83/153	64/101	N.S.
Pathological evaluation	*n* = 13	*n* = 15	
Site of biopsy (BB:QF)	14:6	7:3	N.S.
Inflammation (count of lymphocytes)	1.5 ± 1.1	1.7 ± 0.98	N.S.
Rimmed vacuoles	2.2 ± 1.3	3.2 ± 0.94	*p* < 0.05

mMRC, modified Medical Research Council; MUP, motor-unit potential; N.S., not significant.

All the participants were Japanese nationals. Of the 30 patients, five required a cane for ambulation. None of the patients were bedridden or wheelchair-bound. Characteristics of the study population such as the age during evaluation, age at onset, and disease duration were similar to those reported previously. Elevated creatine kinase (CK) was the most common abnormality in the blood tests. All the patients presented with chronic progressive muscle weakness. The onset symptoms were gait disturbance and difficulty in standing up (*n* = 15), weakness of upper limbs, and difficulty in opening the cap of a plastic bottle (*n* = 15). No patients presented with an initial symptom of dysphagia. Disease onset was defined as the time when muscle weakness began but not when CK was evaluated. The mean serum CK level was 452.7 ± 276.9 (reference value <500 U/mL).

### 3.2 MUPs per muscle

A total of 254 muscles were included in this study (Table 2). We targeted MUPs of 11 muscles, for which 10 or more participants had undergone needle EMG. A high-frequency muscle was defined by an abnormal MUP occurrence rate of 50% or more.

**Table 2:** Abnormal motor-unit potentials between early-and late-stage groups.

	Early			Late		
	Neurogenic suggestive pattern	Myogenic suggestive pattern	*P-*value	Neurogenic suggestive pattern	Myogenic suggestive pattern	*P-*value
Upper						
Deltoid	7.7 (1/13)	7.7 (1/13)	1.000	7.7 (1/13)	15.4 (2/13)	1.0000
Biceps	21.4 (3/14)	42.9 (6/14)	0.4197	8.3 (1/12)	58.3 (7/12)	<0.05
Triceps	9.1 (1/11)	27.3 (3/11)	0.5865	30.0 (3/10)	50.0 (5/10)	0.6499
FDI	15.4 (2/13)	0 0.0 (0/13)	0.4800	8.3 (1/12)	8.3 (1/12)	1.0000
EIP	28.6 (2/7)	0.0 (0/7)	0.4615	12.5 (1/8)	37.5 (3/8)	0.5692
FDP	50.0 (3/6)	83.3 (5/6)	0.5455	20.0 (1/7)	80.0 (6/7)	<0.05
FPL	33.3 (1/3)	66.7 (2/3)	1.0000	20.0 (1/5)	80.0 (4/5)	0.2063
FDS	0.0 (0/4)	25.0 (1/4)	1.0000	25.0 (1/4)	50.0 (2/4)	1.0000
Lower						
VL	42.9 (6/14)	50.0 (7/14)	1.0000	35.8 (5/14)	71.4 (10/14)	0.0642
TA	33.3 (5/15)	60.0 (9/15)	0.2723	28.6 (4/14)	50.0 (7/14)	0.4401
GC	10.0 (1/10)	30.0 (3/10)	0.5820	10.0 (1/10)	40.0 (4/10)	0.3034

All values are shown as percentages (frequency). FDI, first dorsal interosseous muscle; EIP, extensor indicis muscle; FDP, flexor digitorum profundus; FPL, flexor pollicis longus; FDS, flexor digitorum superficialis; VL, vastus lateralis; TA, tibialis anterior; GC, gastrocnemius.

The MUPs of myogenic suggestive pattern in the FDP and flexor pollicis longus (FPL) on EMG were suspicious for IBM, regardless of the disease stage. Myogenic suggestive patterns were more likely to be detected in the biceps, vastus lateralis (VL), and tibialis anterior (TA). No difference was observed in the appearance rate of the neurogenic and myogenic suggestive patterns in the early stage, but the myogenic suggestive pattern appeared more frequently in FDP and biceps in the late stage as compared to the neurogenic pattern. The First dorsal interosseous muscle (FDI) and deltoid muscles had a low frequency of abnormal MUP, with little variation by disease stage.

### 3.3 MUPs of FDP and biceps

**Figure 1:**
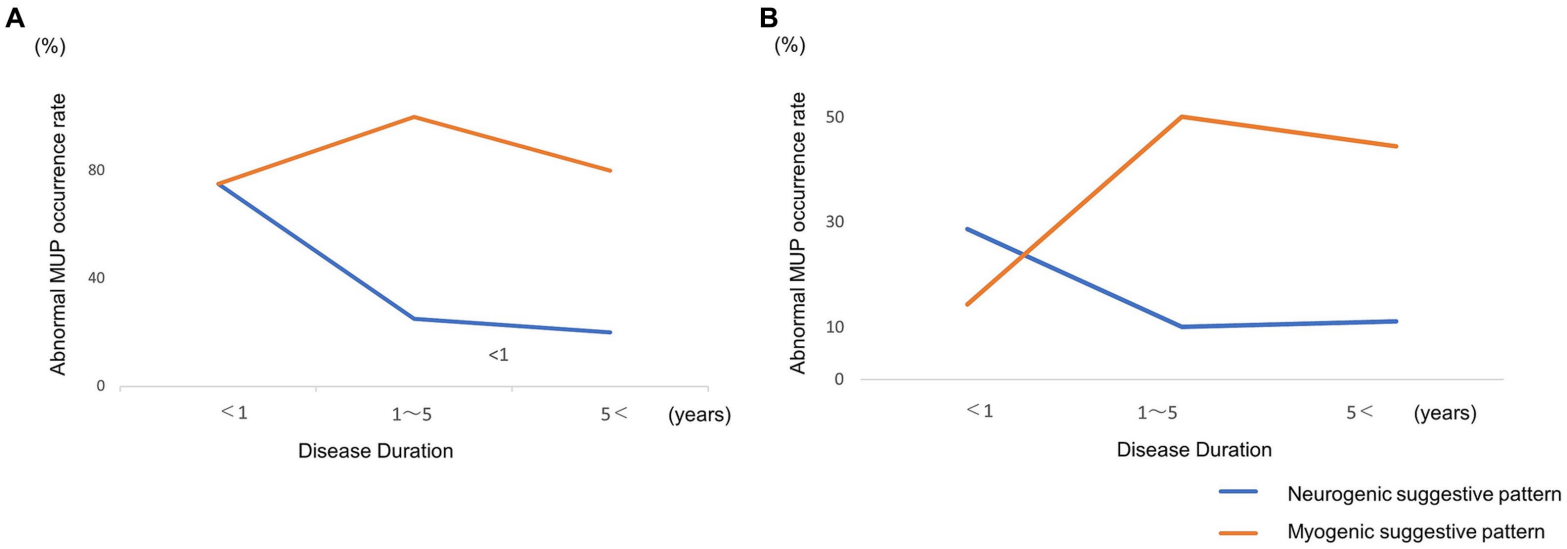
**(A)** Longitudinal changes in abnormal motor-unit potentials of the flexor digitorum profundus. **(B)** Biceps. Neurogenic motor-unit potentials (MUPs) of flexor digitorum profundus (FDP) and Biceps decreased and myogenic MUPs increased over time.

Next, we divided the patients into three groups based on disease duration in the FDP and Biceps: very early (<1 year), middle (1–4 years), and late (5<). For the very early stage, neurogenic suggestive patterns were predominant; however, in the middle and late stage, myogenic suggestive patterns were predominant (Figures 1A,B).

### 3.4 Correlation between muscle strength and MUP pattern

Subsequently, we recorded the EMG of subclinical muscles (i.e., those classified as not being affected by IBM), which were categorized as normal (MRC = 5) or weak (MRC < 5) (Table 3). We used the following six muscles that were available for evaluation of almost separate muscle strength: deltoid, biceps, triceps, VL, TA, and gastrocnemius. Abnormal MUP was observed even in the muscles without muscle weakness, and no difference was observed between neurogenic and myogenic suggestive patterns. Notably, abnormal MUPs increased as the muscle weakness progressed. Biceps and Gastrocnemius with muscle weakness showed a myogenic suggestive pattern rather than a neurogenic one. This result showed that the MUP abnormalities of EMG appeared before muscle weakness.

**Table 3:** Abnormal motor-unit potentials between normal muscle strength and weakness.

	mMRC >5			mMRC <5		
	Neurogenic suggestive pattern	Myogenic suggestive pattern	*P-*value	Neurogenic suggestive pattern	Myogenic suggestive pattern	*P-*value
Upper						
Deltoid	0.0 (0/17)	0.0 (0/17)	1.0000	22.2 (2/9)	33.3 (3/9)	1.0000
Biceps	7.7 (1/13)	23.1 (3/13)	0.5930	23.1 (3/13)	84.6 (11/13)	<0.01
Triceps	0.0 (0/11)	18.1 (2/11)	0.4762	40.0 (4/10)	60.0 (6/10)	0.6563
Lower						
VL	20.0 (2/10)	70.0 (7/10)	0.0698	50.0 (9/18)	55.6 (10/18)	1.0000
TA	22.2 (4/18)	16.7 (3/18)	1.0000	45.4 (5/11)	81.8 (9/11)	0.1827
GC	6.7 (1/15)	13.3 (2/15)	1.0000	20.0 (1/5)	100.0 (5/5)	<0.05

All values are shown as percentages (frequency). mMRC, modified Medical Research Council; VL, vastus lateralis; TA, tibialis anterior; GC, gastrocnemius.

### 3.5 MUP pattern of the contralateral side of the biopsy muscle and pathological findings

Twenty-two muscles with biopsies from the same muscle, contralateral to the muscle in which electromyography was performed, were analyzed. MUP of electromyogram showed that 12 muscles were myogenic, three were neurogenic, and seven were within the normal range. Comparing the pathological findings of 12 myogenic muscles and 10 normal and neurogenic muscles, the inflammatory cells were 1.8 + 1.0, 1.2 + 0.42 (*p* = 0.071) and rimmed vacuoles were 3.25 + 0.97, 2.3 + 1.3 (*p* = 0.067). Although no significant difference was observed, there was a tendency for more inflammatory cells and RV on the contralateral side of the muscle, which exhibited a myogenic suggestive pattern.

## 4 Discussion

IBM diagnosis is often delayed by an average of 5 years after symptom onset. It is diagnosed using a combination of clinical, neuroelectrophysiological, and pathological evaluations (10). In clinical practice, EMG results are central to the diagnosis and selection of patients for muscle biopsy (1). However, EMG findings vary with the stage of the disease and the extent of muscle damage. Clinicians should understand their characteristics to interpret EMG findings by stage.

IBM EMG has mixed myogenic and neurogenic patterns, and the findings are considered non-specific (11, 12). Our research also confirmed two patterns. In this study, EMG findings confirmed the difference in the frequency of occurrence between myogenic and neurogenic suggestive patterns of biceps and FDP in late stages. Conversely, no difference was observed in the initial stage of the two muscles. This result suggests that as the disease progresses, myogenic changes become the main causative factor. In FDP, neurogenic suggestive patterns are conspicuous within 1 year of onset, suggesting redistribution due to degeneration before onset. In cases where more than a year has passed, redistribution may not have been completed. The FDI and deltoid activities did not suggest myogenic changes over time. Moreover, myogenic changes tended to occur in the distal muscles, whereas neurogenic changes tended to coexist in the proximal muscles even during persistent muscle weakness. In the lower-limb muscles, the VL and TA showed myogenic suggestive patterns from the early stages, but some neurogenic suggestive patterns were also observed. Neurogenic suggestive patterns predominated in the muscles of the lower extremities and when there was muscle weakness. Hence, lower-extremity muscles should not be judged based on MUPs alone but also in conjunction with other findings, such as a poor recruitment pattern. In this study, we identified long-lasting or neurogenic suggestive patterns of MUPs as an evidence of a neuropathic component of IBM (13). Long-term polyphasic MUPs in myopathies correlated with fiber regeneration (14). Additionally, the high amplitude is thought to be caused by residual muscle fiber hypertrophy. Neurogenic changes in EMG likely represent a signature of denervation in IBM and segmented muscle fiber reinnervation. We observed a tendency for myogenic formation to increase in the early and late stages. However, the rate of neurogenic changes did not change. Although neurogenic changes are considered as the effects of aging or other coexisting evidence, it is possible that advanced myogenic changes in the late stage were mistakenly interpreted as neurogenic changes.

Our results showed that abnormal MUPs are observed even in muscles with normal strength. This suggests that the disease state appears before the awareness of muscle weakness and that by the time muscle weakness appears, the disease state has already progressed. IBM progresses slowly; thus, we observed long-lasting neurogenic suggestive pattern MUPs caused by reinnervation. The action potentials in neurogenic motor units of IBM are sufficiently dense to overshadow myogenic changes. Moreover, MUPs are often complex in morphology, mimicking subacute neuropathic processes (15, 16). MUP morphology not only represents neurogenic and myogenic activity but is also a tool for expressing developmental progression and degree. The characteristics of MUPs reflect the complexity of underlying disease mechanisms.

Originally, IBM was considered a primary inflammatory myopathy based on endomyositis; however, its etiological contribution, such as whether it is pure myopathy, remains unclear. Some studies reported about peripheral neuropathy in IBM (17, 18). Subjective symptoms and neurological examinations do not suggest sensory deficits; nonetheless, abnormalities may be observed in a nerve conduction study (NCS), which may indicate nerve fiber damage within the muscle. Axonal loss and reduced mean corpuscular volume amplitude is suggestive of Wallerian degeneration and atrophy of axon terminals, while delayed F-wave latency is suggestive of peripheral nerve involvement in IBM (17). Another study reported that 30% of patients with IBM had decreased NCS conduction velocities (12). Using muscle biopsy, IBM is characterized by a unique combination of inflammatory cells, rimmed vacuoles, and protein aggregation. Muscle nuclear degeneration occurs early in IBM when fringed vacuoles mostly comprise of nuclear membrane proteins. Nuclei are abnormally filled with neurofilaments, which is an early detectable pathological hallmark (12). In this study, the presence of rimmed vacuoles was higher in the late stage than that in the early stage; however, the number of inflammatory cells remained unchanged. The biceps and quadriceps, which were the target muscles of the biopsy, showed myogenic changes even at the late stage in EMG, and the rimmed vacuoles may represent muscle degeneration. Contralateral electromyography findings may be helpful in selecting muscles for muscle biopsy, such as the biceps and quadriceps. Our study targeted patients diagnosed pathologically with IBM. However, this study had some limitations. First, important muscles, such as the FDP and VL, were not examined in all the cases. In addition, we selected a small number of cases because they were confirmed pathologically. FPL also tended to show a myogenic pattern in the later stages, and increasing the number of cases included in the study may increasing the number of cases included in the study may help in achieving greater accuracy. Second, we defined myogenic suggestive pattern or neurogenic by only the duration. A comprehensive evaluation of interference waves, recruitment, etc., should be performed to differentiate the two patterns. Each muscle has its own set of MUP characteristics, such as a lower amplitude in the biceps and higher amplitudes in lower-extremity muscles. Third, we could not analyze the genes that have been reported in some cases.

EMG findings vary with the stage of IBM and the extent of muscle damage. Clinicians should understand their characteristics to interpret EMG findings according to the stages.

## Data availability statement

The raw data supporting the conclusions of this article will be made available by the authors, without undue reservation.

## Ethics statement

The studies involving humans were approved by the Nara Medical University Hospital. The studies were conducted in accordance with the local legislation and institutional requirements. Written informed consent for participation was not required from the participants or the participants’ legal guardians/next of kin in accordance with the national legislation and institutional requirements.

## Author contributions

TM: Conceptualization, Data curation, Funding acquisition, Investigation, Writing – original draft, Writing – review & editing. NIg: Data curation, Writing – review & editing. NIw: Data curation, Writing – original draft. NY: Data curation, Writing – original draft. HH: Data curation, Writing – original draft. KS: Supervision, Writing – review & editing. NE: Data curation, Writing – review & editing.
